# Does electrical stimulation synchronized with ankle movements better improve ankle proprioception and gait kinematics in chronic stroke? A randomized controlled study

**DOI:** 10.3233/NRE-220018

**Published:** 2022-09-30

**Authors:** Ji-Eun Cho, Joon-Ho Shin, Hogene Kim

**Affiliations:** a Department of Rehabilitative and Assistive Technology, National Rehabilitation Center, Seoul, South Korea; b Department of Rehabilitation Medicine, National Rehabilitation Center, Seoul, South Korea; c Department of Clinical Rehabilitation Research, National Rehabilitation Center, Seoul, South Korea

**Keywords:** Ankle, electrical stimulation, proprioception, range of motion, stroke

## Abstract

**BACKGROUND:**

Individuals with stroke have impaired sensorimotor function of ankle.

**OBJECTIVE:**

To investigate the effects of passive biaxial ankle movement training synchronized with electrical stimulation therapy (AMT-EST) on ankle proprioception, passive range of motion (pROM), and strength, balance, and gait of chronic stroke patients.

**METHODS:**

Thirty-five stroke patients were randomized. The experimental group received a total of 20 AMT-EST sessions. The control group received only EST. Primary outcome measures were ankle functions. Secondary outcome measures were clinical assessments of motor, balance, and gait-related functions. All assessments were compared before and after the intervention.

**RESULTS:**

The experimental group had significantly improved ankle dorsiflexor strength (*p* = 0.015) and ankle pROM during foot supination (*p* = 0.026) and pronation (*p* = 0.004) and clinical assessment (Fugl–Meyer Assessment of the lower extremities [FM-L], Berg Balance Scale, Timed Up and Go test, Fall Efficacy Scale, walking speed, and step length; all *p* < 0.05) values. The regression model predicting ankle proprioception showed significantly large effects (adjusted R^2^ = 0.493; *p* < 0.01) of the combined FM-L score and time since stroke.

**CONCLUSION:**

Biaxial AMT-EST resulted in better ankle pROM and strength than conventional EST. Ankle proprioception was not significantly improved after AMT-EST and was predicted by the FM-L score and time since stroke.

## 1 Background

Somatosensory impairments are observed in up to 89%of stroke survivors (Connell, Lincoln, & Radford, 2008) and are more prominent in the lower extremities than in the upper extremities (Tyson, Hanley, Chillala, Selley, & Tallis, 2008). Disabled sensory inputs alter normal cortical representations within the sensory cortex and motor cortex and cause changes in motor performances, potentially contributing to balance and gait asymmetry (Tyson, Hanley, Chillala, Selley, & Tallis, 2006; Wutzke, Mercer, & Lewek, 2013). Moreover, a direct relationship between walking function and impaired light touch sensation and the proprioceptive acuity of the ankle of individuals who have experienced stroke has been observed (Lee, Kilbreath, & Refshauge, 2005).

Various somatosensory inputs have been used as therapeutic interventions to enhance motor recovery of the lower extremities of patients with neurological impairments. For example, electrical stimulation therapy (EST) aims to achieve sensorimotor integration, thereby ensuring better lower extremity function and pain control in individuals with paraplegia (Benton, 1981; Leung & Moseley, 2003). Combined therapy with EST and other modalities has been found to modulate ankle spasticity (Cheng, Yang, Cheng, Lin, & Wang, 2010), improve functional ambulation outcomes (Cheng et al., 2010; Tong, Ng, Li, & So, 2006), clinical gait characteristics (Cheng et al., 2010; Kesar et al., 2011; Tong et al., 2006), and enhance the recovery of physical conditions (Peng et al., 2011) of stroke survivors. A meta-analysis showed that EST contributes to conventional rehabilitation therapies and improves lower extremity motor function without increasing spasticity (Sharififar, Shuster, & Bishop, 2018). Moreover, combined EST and task-related training has demonstrated decreased plantar flexor spasticity, improved dorsiflexor and plantar flexor strength, and increased gait velocity more than EST alone, task-related training, and no treatment (Ng & Hui-Chan, 2007). Therefore, it has been observed that EST combined with functional movements induces a sensory cue to activate motor neurons or reflex pathways via stimulation of sensory nerve fibers (Popovic & Sinkjaer, 2003), thereby increasing the strength of afferent inputs to promote motor learning (Schuhfried, Crevenna, Fialka-Moser, & Paternostro-Sluga, 2012).

Passive range of motion (pROM) exercise aims to maintain or increase joint mobility by influencing the extensibility of the lower motor neurons as well as soft tissues, thereby reducing spasticity and directly or indirectly increasing muscle extensibility (Wiles & Stiller, 2010). Moreover, pROM provides sensory information derived from muscle spindles, Golgi tendon organs, and joint/cutaneous receptors (Lephart, 2000; Riemann & Lephart, 2002). One study demonstrated that repeated feedback-controlled intelligent stretching of dorsiflexors and plantar flexors of the ankle joint affected the pROM, strength, ankle stiffness, and comfortable walking speed without significant changes in the active ROM, energy loss, and excitability of the ankle after stroke (Selles et al., 2005). A recent study reported that 4 weeks of passive biaxial ankle training for chronic stroke patients improved ankle stiffness, ankle pROM, and walking performance on uneven surfaces (Kim, Cho, & Lee, 2019). However, most studies focused on ankle motor function only. There are few studies of the enhancement of ankle sensory functions after EST. Therefore, EST as an adjunct to ankle biaxial pROM exercise can effectively provide sensory information to hemiparetic stroke patients who have difficulty with voluntary ankle control.

This study aimed to investigate the effects of passive biaxial ankle movement training (AMT) synch-ronized with EST (AMT-EST) on ankle proprioception, pROM, strength, and functional performance, including lower extremity impairment, balance, and gait. We hypothesized that AMT-EST would more significantly improve the ankle sensorimotor function of chronic stroke patients than of individuals in the control group. We also hypothesized that this ankle training would improve the functional abilities associated with the ankle joint, including motor, balance, and gait functions.

## 2 Methods

### 2.1 Design

A double-blind, parallel-group, randomized, controlled trial with blinded assessors and concealed allocation was conducted. An individual not involved in the trial performed blocked random allocation of 35 participants using Microsoft Excel^®^, and participants were divided accordingly into the experimental group or control group. Participants were blinded to their allocation, and all assessments and interventions were performed by a physical therapist who was not involved in the study. All participants were assessed before the group allocation and reassessed at the end of the 4-week intervention period.

### 2.2 Participants

Inpatients at a rehabilitation hospital were re-cruited as participants from January to May 2018. The eligibility criteria were as follows: chronic poststroke hemiparesis; weakness of the ankle muscles on the affected side (Medical Research Council Scale ankle dorsiflexion [DF] strength grades 1–3); Modified Ashworth Scale score < 3 for spasticity for the affected ankle; impaired proprioception of the affected foot; and Functional Ambulatory Category score≥3. Potential participants were excluded for the following reasons: complications of orthopedic disorders such as ankle contracture and fracture; unwillingness to receive EST; and cognitive impairment (Mini-Mental State Examination score≤24). This study was approved by the Institutional Review Board of a rehabilitation hospital, and participants provided written informed consent before study enrollment.

### 2.3 Ankle movement training system

The ankle training device used during this study was developed for intensive and selective ankle training for stroke patients. The main features of the ankle training device were that it could reproduce the actual biaxial ankle movement that was applied by a seesaw-type foot cradle that pivoted along the transverse ankle axis, and it had a foot force plate that was rotated along a 42-degree tilted subtalar axis relative to the foot cradle. During this study, the training protocol that applied EST in accordance with passive biaxial ankle movement was constructed and applied (see the Appendix).

### 2.4 Intervention

#### 2.4.1 Experimental group

**Fig. 1:**
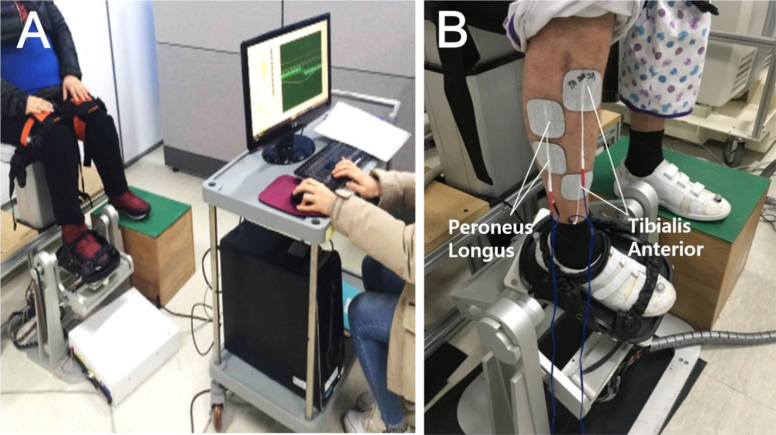
Before ankle movement training with electrical stimulation, the participants sat on a height-adjustable chair and the paretic foot was fastened to the force plate of the ankle training device (A). The two electrode pairs were placed on the motor point of the tibialis anterior to induce ankle dorsiflexion and on the peroneus longus to induce ankle eversion (B).

Before the training session, participants were asked to comfortably sit on a height-adjustable chair with the knees flexed at 90 degrees, to place the paretic foot on the footplate of the ankle training device, and to place the nonparetic foot on the height-matched footrest. The paretic foot was fastened to the force plate in the foot cradle using three length-adjustable straps (Fig. 1). The two electrode pairs (5×9 cm; RehaTrode, Hasomed, Germany) were placed over the common peroneal nerve as it passed over the head of fibula and the motor point of the tibialis anterior. Other electrode pairs were placed slightly lateral to this and targeted toward the peroneus longus (Fig. 1). Electrical stimulation was applied to confirm that the location of the attached electrodes caused proper ankle dorsiflexor (tibialis anterior) and evertor (peroneus longus).

**Fig. 2:**
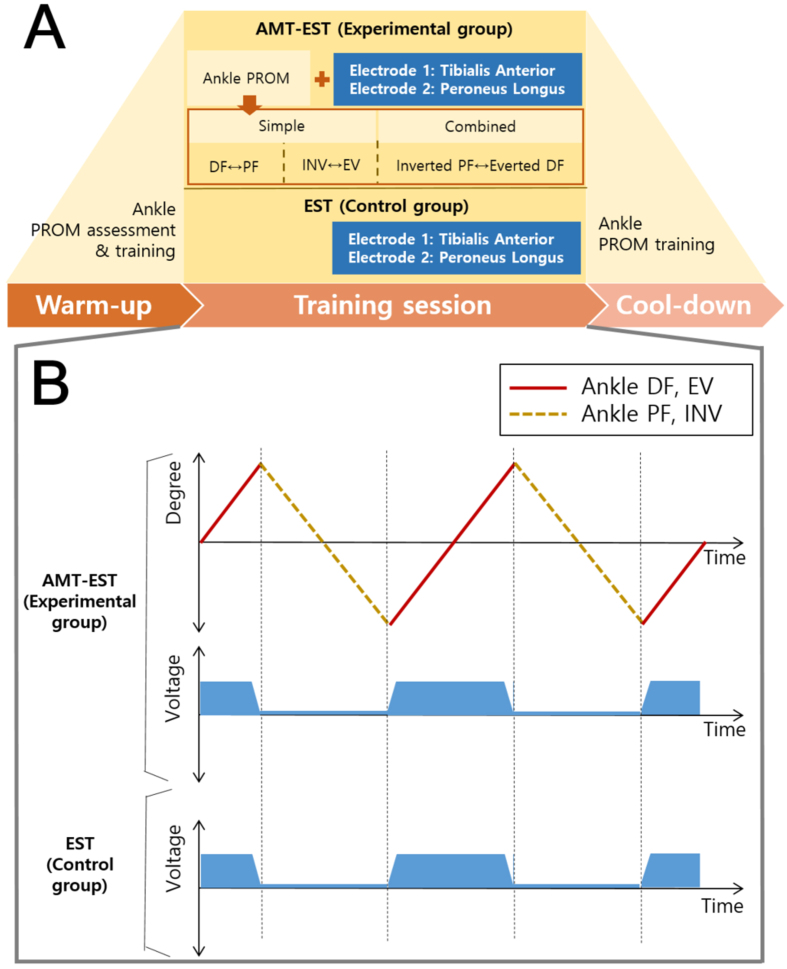
Ankle training consisted of a warm-up session (5 min), training session (30 min), and cool-down session (5 min). The experimental group received AMT-EST comprising single-movement training and combined-movement training. The control group received only electrical stimulation (A). Electrical stimulation was applied only during ankle dorsiflexion and pronation (B). AMT-EST, ankle movement training with electrical stimulation therapy; EST, electrical stimulation therapy; DF, dorsiflexion; PF, plantar flexion; SN, supination; PN, pronation.

AMT-EST was performed for 4 weeks (one 40-minute session per day, 5 days per week). All participants completed 100%of the training sessions. The three steps of the ankle pROM exercise with EST were performed along the ankle (talocrural) and subtalar (talocalcaneal) axes for 40 minutes (Fig. 2). The first step consisted of 20 repetitions of DF-plantarflexion (PF), the second step consisted of 20 repetitions of supination (SN)-pronation (PN), and the third step consisted of 40 repetitions of a larger ROM of SN-PN (more diagonal movements). All ankle movement speed was slow (2.14 degrees/second) because fast stretching is a strong stimulation and will elicit a more powerful reflex contraction (Liebesman & Cafarelli, 1994) The timing of starting and the timing of ending the paretic ankle DF and PN movements were directly observed by the therapist, who then applied EST (Microstim2; Model GmbH, Germany) with 0.28-ms pulses at 35 Hz with pulse durations from 300 to 450μs in alternating mode within the participant’s tolerance level via surface electrodes. The amplitude was adjusted to produce muscle contractions without causing patient discomfort (Burridge & Ladouceur, 2001).

#### 2.4.2 Control group

Participants in the control group received EST for the paretic ankle muscles for 4 weeks (5 sessions per week). EST (Microstim2; Model GmbH, Hamburg, Germany) with a pulse frequency of 35 Hz in alternating mode and pulse duration of 300 to 450μs was applied with the electrodes in the same position as that used for the experimental group in the sitting position. In addition, the inpatient rehabilitation program for stroke patients in our center was identically provided to both the experimental and control groups, which included muscle strengthening exercises, gait training, and the occupational therapy for 90 min/day, 5 days/week, for 4 weeks.

### 2.5 Outcome measures

The primary outcome was ankle function, including ankle proprioception, pROM, and strength. Proprioception was assessed by evaluating the joint proprioception of the ankle DF, PF, SN, and PN using an ankle training device with constant velocities (2.14 degrees/second). Participants wore eye masks and earplugs while in the sitting position with the lower limbs fixed to allow only ankle movement. The assessment comprised two steps. During the first step, the ankle was moved passively from the initial angle (0 degrees) to the randomly assigned 10 target angles (10 degrees, 20 degrees, and 30 degrees of ankle PF and SN and 10 degrees and 20 degrees of ankle DF and PN according to the normal ROM of the ankle) while asking the participant whether the ankle movement and the direction of movement were perceivable. After staying at the target position for 5 seconds, the ankle was returned to the initial angle. During the second step, the paretic ankle was moved toward the target angle again, and the participant was asked to say “stop” when the target angle (actual angle) was reached. No feedback about results was provided to the participant during the task. The assessment began with a period of familiarization. Three ankle movements were evaluated in each direction, and a total of 38 measurements including dummy trials (no movement) were performed. For statistical analyses, the proprioception ratios were calculated in relation to angular differences (the differences between the target angle and actual angle) (Contu et al., 2017). Finally, the proprioception ratios for all four directions (DF, PF, SN, and PN) were calculated as the average value of the proprioception ratio that was measured three times for each angle. The larger the proprioception ratio value, the greater the deficit.

$$\begin{array}{cc} & \mathrm{Ankle}\mathrm{proprioception}=\\ & \;\frac{\begin{array}{c} 10\;\mathrm{degrees}\\ \mathrm{ratio} \end{array}+\begin{array}{c} 20\;\mathrm{degrees}\\ \mathrm{ratio} \end{array}+\begin{array}{c} 30\;\mathrm{degrees}\\ \mathrm{ratio} \end{array}}{3} \end{array}$$

The pROM of the paretic ankle was measured by a skilled physiotherapist using a portable goniometer. The average values of the three measurements of the maximum pROM of DF, PF, PN, and SN were recorded. To measure ankle strength, the maximal voluntary isometric contraction force of the paretic ankle muscle was measured using a portable manual muscle strength tester (Lafayette Instrument, Lafayette, IN, USA). The participants were in the sitting position during the measurements, and resistance was provided using a measurer to isolate the ankle joint motion (DF, PF, PN, and SN). Prior to each trial, maximum voluntary isometric contraction was measured by holding for at least three seconds during maximal efforts of target muscle contraction without visible joint movements and any concentric and eccentric muscle contractions; a 30-second rest period was provided between trials. The average value was used for analysis.

Secondary outcomes included motor, balance, and gait functions. Clinical assessments were performed using the Fugl–Meyer Assessment for the lower extremities (FM-L), Berg Balance Scale (BBS), the Timed Up and Go test (TUG), the Korean version of the Fall Efficacy Scale, and walking speed to evaluate motor and balance functions (see Appendix).

During gait evaluations, the subjects repeated walking trials at least four times on a 1.5- x 10-m walkway covered with industrial carpeting at a comfortable speed. The kinematic data were recorded using a VICON motion analysis system (VICON, Saint Helens, UK) and sampled at 100 Hz with 24 reflective passive markers following a plug-in gait model. The kinematic data were processed by Visual 3D (C-Motion, Germantown, MD, USA) using a fourth-order zero-lag Butterworth low-pass filter with 6-Hz cutoff frequencies. The step kinematics on the paretic side were processed to acquire the walking speed at the body center of mass, step length, step time, and step width. The joint kinematics were postprocessed to calculate the ROM of hip flexion/extension, knee flexion/extension, ankle DF/PF, and ankle SN/PN on the paretic side.

### 2.6 Sample size calculation

The sample size was calculated as previously described by a study that reported repeated passive and active exercises for patients with hemiplegia. The calculation was based on the paired *t*-test value of knee proprioception after the repeated passive exercises (Kwon & Lee, 2013) using an acceptable level of significance of 0.05 at 95%power. The total sample size was determined to be six for each group (expected effect size: 1.744; actual power: 0.975). In one study (Ng & Hui-Chan, 2007) that that showed the effectiveness of the similar task-related ankle training, e.g. ankle strengthening after electrical stimulation, the total sample size was calculated and determined to be 10 for each group in order to conduct the paired *t*-test (expected effect size: 1.166; actual power: 0.959). Considering the power of analysis, the variables, and a 20%dropout rate that could occur during the research process, a total of 35 participants (18 in the experimental group and 17 in the control group) were recruited for the study. The G^*^ Power 3.1.9.2 program was used.

### 2.7 Statistical analyses

Before all analyses, the normality of data was assessed using the one-sample Kolmogorov-Smirnov test. Comparisons of the changes between the two groups were performed using the Mann-Whitney *U* test, and changes within groups were compared using the Wilcoxon signed rank test. A stepwise conditional multiple regression analysis was used to examine the relationship between ankle proprioception (dependent variable) and other outcomes (independent variables). Spearman’s rank-order correlations (Spearman’s rho) were used to check for multicollinearity between independent variables; variables of significant correlation with ankle proprioception (*p* > 0.05) were used during the regression analysis. Threshold values for the interpretation of the adjusted R^2^ as an effect size were set at 0.02 (small), 0.13 (medium), and 0.26 (large) in accordance with the work of Cohen (Cohen, 1988). All statistical analyses were performed using SPSS version 22.0 (IBM, Armonk, NY, USA), and the level of significance was set at *P* < 0.05.

## 3 Results

### 3.1 Participants

Five individuals in the control group dropped out of the study due to their personal and administrative reasons. Three of them transferred to another hospital without completing their training sessions due to acute health issues such as falls, and the other two participants failed to extend their discharge date as planned. Therefore, the postintervention testing and analysis were completed for 18 individuals in the experimental group and 12 individuals in the control group. A CONSORT diagram is presented in the Appendix. Baseline characteristics of the participants are presented in Table 1. At baseline, no significant between-group differences in demographics or measurements were observed.

**Table 1:** Baseline characteristics of the experimental and control groups

	Experimental group (*n* = 18)	Control group (*n* = 12)	*P*-value
Age (years)	51.8 (12.0)	55.0 (10.9)	0.589^†^
Sex (m/f)	14/4	9/3	0.798^*^
Weight (kg)	69.6 (9.4)	70.6 (12.2)	0.982^†^
Height (m)	169.8 (7.7)	169.6 (8.9)	0.893^†^
Time post stroke (months)	11.6 (4.1)	8.6 (3.9)	0.065^†^
Stroke side (r/l)	9/9	5/7	0.815^*^
Modified Ashworth Scale (0/1/1 + /2)	(0/5/12/1)	(1/4/7/0)	0.436^*^
Functional ambulation category (0–5)	4.4 (1.0)	4.3 (0.6)	1.000^†^
K-MMES score	26.8 (2.5)	28.4 (1.1)	0.114^†^

Values are expressed as mean (SD) unless otherwise stated. K-MMES: Korean Version of the Mini-Mental State Examination. ^*^Chi-square test. ^†^Mann–Whitney *U* test.

### 3.2 Primary outcomes: Ankle proprioception, passive range of motion, and strength

The specific ankle function values before and after training are presented in Table 2 and in the Appendix. After completing the 20 training sessions, all ankle proprioception in both the experimental and control group decreased, with no significant changes (*P* > 0.05). However, the ankle pROM of SN and PN showed significant improvements in the experimental group only (*P* < 0.05), and it was significant during between-group comparisons (*P* < 0.05). Moreover, the ankle strength of DF, PF, SN, and PN showed significant improvement in the experimental group only (*P* < 0.05). In particular, the experimental group showed significantly more changes in the strength of ankle DF than the control group (*P* = 0.015).

**Table 2:** Outcome values of ankle function before (pre) and after (post) the 4-week treatment (*N* = 30)

Measures (unit)	Experimental group (*n* = 18)			Control group (*n* = 12)			Between group
		Pre	Post	*P*-value	Pre	Post	*P*-value	*P*-value
Proprioception (%)	Dorsiflexion	61.1 (37.0)	59.4 (34.0)	0.605	40.4 (30.3)	40.0 (32.5)	0.799	0.621
	Plantarflexion	60.6 (36.0)	51.3 (33.7)	0.062	35.0 (27.0)	33.6 (29.0)	0.477	0.150
	Supination	57.2 (35.8)	48.4 (29.2)	0.085	38.1 (32.0)	31.9 (23.3)	0.594	0.529
	Pronation	65.8 (35.3)	56.8 (34.5)	0.109	37.5 (29.3)	33.6 (26.6)	0.109	0.345
pROM (°)	Dorsiflexion	12.2 (6.2)	14.7 (7.5)	0.226	15.7 (6.3)	11.6 (7.1)	0.161	0.058
	Plantarflexion	44.7 (10.1)	45.2 (8.9)	0.981	41.3 (8.8)	40.9 (9.7)	0.656	0.857
	Supination	21.1 (4.5)	24.3 (4.7)	**0.029** ^*^	24.5 (2.9)	23.0 (2.5)	0.307	**0.026** ^*^
	Pronation	19.2 (4.3)	23.0 (3.8)	**0.003** ^†^	21.0 (3.9)	19.3 (4.7)	0.259	**0.004** ^†^
Strength (N)	Dorsiflexion	10.8 (3.7)	16.4 (4.6)	**0.001** ^†^	13.6 (3.9)	13.7 (6.4)	0.964	**0.015** ^*^
	Plantarflexion	14.9 (6.5)	18.2 (5.7)	**0.036** ^*^	14.3 (3.3)	15.0 (5.7)	0.755	0.157
	Supination	7.7 (3.2)	10.7 (2.1)	**0.002** ^†^	9.1 (2.1)	9.7 (2.6)	0.473	0.058
	Pronation	7.2 (3.2)	9.8 (2.2)	**0.007** ^†^	7.8 (1.2)	8.5 (2.1)	0.437	0.161

Values are expressed as means (SD) unless otherwise stated. pROM: passive range of motion. ^*^P < 0.05. ^†^*P *< 0.01.

### 3.3 Secondary outcomes: Functional abilities related to motor, balance, and gait functions

The specific values for motor, balance, and gait function variables before and after training are presented in Table 3. After the training session, the experimental group had significant improvements in the FM-L, BBS, TUG, and Fall Efficacy Scale values (*P* < 0.05). The control group had significant improvements in the BBS and TUG values after training (*P* < 0.05). Regarding the gait-related variables, the experimental group had significant improvement in walking speed and step length (*P* < 0.05). In the control group, only step length showed significant improvement after training (*P* < 0.05). No significance was observed during the between-group comparison.

**Table 3:** Motor, balance, and gait related outcome values before (pre) and after (post) the 4-week treatment (*N* = 30)

Measures (unit)		Experimental group (*n* = 18)			Control group (*n* = 12)			Between group
		Pre	Post	*P*-value	Pre	Post	*P*-value	*P*-value
*Motor and balance assessments*								
FM-L (score)		17.8 (3.3)	22.4 (3.5)	**< 0.001** ^†^	18.6 (2.9)	20.6 (3.3)	0.113	0.082
Berg balance scale (score)		46.2 (6.1)	49.6 (4.7)	**< 0.001** ^†^	45.0 (9.1)	47.0 (6.7)	**0.035** ^*^	0.166
Timed up and go (sec)		33.1 (16.2)	28.0 (14.9)	**0.011** ^*^	40.1 (18.7)	34.3 (19.0)	**0.023** ^*^	0.759
Fall Efficacy Scale (score)		53.6 (30.6)	31.7 (18.2)	**0.003** ^†^	53.8 (27.8)	50.6 (33.6)	0.824	0.157
*Gait kinematics*
Walking speed (cm/s)		34.5 (19.3)	41.0 (22.1)	**0.015** ^*^	33.0 (22.1)	37.0 (26.2)	0.093	0.564
Step length (cm)		34.0 (10.5)	38.5 (10.6)	**0.019** ^*^	27.5 (17.6)	33.8 (16.5)	**0.037** ^*^	0.815
Step time (msec)		529.4 (92.0)	506.0 (89.9)	0.206	545.5 (150.7)	536.4 (120.6)	0.785	0.172
Step width (cm)		17.9 (3.2)	18.5 (3.4)	0.594	15.4 (6.7)	17.3 (5.0)	0.878	0.703
Joint ROM (°)	Ankle DF-PF	21.4 (8.2)	23.4 (7.7)	0.105	20.9 (13.5)	22.9 (13.5)	0.155	1.000
	Ankle SN-PN	4.7 (1.6)	4.7 (1.4)	0.842	9.1 (5.9)	9.7 (6.1)	0.173	0.356
	Knee Flx-Ext	43.9 (15.7)	46.9 (13.9)	0.408	32.1 (16.9)	35.0 (18.1)	0.398	1.000
	Hip Flx-Ext	32.3 (10.5)	34.4 (10.9)	0.127	26.8 (12.9)	28.8 (13.8)	0.131	0.693
	Hip Abd-Add	9.2 (2.4)	10.5 (2.9)	0.083	9.0 (4.4)	10.4 (3.8)	0.056	0.767

Values are expressed as means (SD) unless otherwise stated. FM-L: Fugl–Meyer Assessment of the lower extremities, ROM: range of motion, DF-PF: dorsiflexion-plantarflexion, SN-PN: supination-pronation, Flx-Ext: flexion-extension, Abd-Add: abduction-adduction. ^*^P < 0.05. ^†^*P* < 0.01.

### 3.4 Relationship between ankle proprioception and other outcome measures at baseline

Ankle proprioception was significantly correlated with time after stroke, weight, pROM of PN, BBS score, FM-L score, and step width (*P* < 0.05). Finally, the FM-L score and time after stroke were combined in a significant regression model to predict ankle proprioception (adjusted R^2^ = 0.493; large effect) (Table 4).

**Table 4:** Results of multiple regression analysis between baseline ankle proprioception and other outcomes

Variables	B	SE	β	T	P
(Constant)	113.048	38.848	–	2.910	0.008
FM-L	–5.081	1.661	–0.494	–3.059	0.006
Time post stroke (months)	3.091	1.311	0.381	2.358	0.028

FM-L: Fugl–Meyer Assessment of the lower extremities. R^2^ =0.537, Adju-R^2^ = 0.493, *F* = 12.166, *P* < 0.001. Regression equation: Ankle proprioception = 113.048–5.081^*^FMA + 3.091^*^Time post stroke.

## 4 Discussion

This study demonstrated that AMT-EST was more effective than conventional EST without movements for improving ankle function, including pROM of SN and PN, and strength of DF of chronic stroke patients. After AMT-EST, participants showed improvements in motor function, balance, gait speed, step length, and ankle function. Although ankle proprioception did not show significant improvement after training, the result of the regression analysis postulated that ankle proprioception was affected by motor impairment (FM-L score) and time since stroke for chronic stroke patients.

The ankle sensory function is considered important in the recovery of lower limb function of stroke patients. Proprioception provides self-information regarding position, movement, or force necessary for the body to make motor function adjustments (Simoneau, Ulbrecht, Derr, & Cavanagh, 1995). Recent studies have found that ankle proprioceptive deficits have significant relationships with mobility, balance, balance confidence, physical functions, and activities of daily living (Deshpande et al., 2016; Rand, 2018). We found that ankle proprioception was significantly related to weight, time since stroke, pROM of ankle PN, BBS score, FM-L score, and step width. Additionally, the regression analysis results explained 50%of ankle proprioception with regard to lower limb motor function and time since stroke. The sensory system has an important role in both feedforward and feedback operations to achieve novel motor learning and motor adjustment (Shumway-Cook & Woollacott, 2007). Therefore, interventions to improve sensory input potentially affect motor control, balance, and daily functioning (Berthoz, 2000).

AMT-EST was intended to provide enhanced sensory input by combining electrical stimulation with biaxial ankle movements. The electrical stimulation combined with functional motion has been reported to further enhance afferent inputs to promote motor performance (Laufer, Ring, Sprecher, & Hausdorff, 2009). It may enhance the generation of cortical brain perfusion to the ipsilesional sensorimotor cortex (Hara, Obayashi, Tsujiuchi, & Muraoka, 2013). In a previous study, the application of electrical stimulation to ankle muscles during gait and everyday activities significantly increased ankle proprioception, ankle strength, balance, and gait speed (Tyson, Sadeghi-Demneh, & Nester, 2013). Other reviews have suggested that electrical stimulation combined with active training may facilitate motor recovery (Laufer & Elboim-Gabyzon, 2011). Nevertheless, we performed passive ankle training because most of the stroke patients in this study were unable to selectively control the ankle joint. This repeated pROM training provides sensory input for muscle extensibility, which is responsible for the maintenance of stretch receptors of the muscle spindle. Passive biaxial AMT effectively promotes ankle proprioception that recognizes the positional sense of joints with regard to changes in muscle length (Fortier & Basset, 2012). Similarly, this study demonstrated that AMT-EST effectively increased the strength of ankle muscles as well as the pROM of SN and PN. This suggests that enhancement of ankle proprioception may potentially affect ankle strength, which comprises motor performance. Another study reported that repeated passive biaxial ankle training for chronic stroke patients affected ankle stiffness, ankle pROM, and walking performance on uneven surfaces (Kim et al., 2019). These results could potentially infer that there was a change in the ankle motor function and ankle sensory function related to sensory input caused by changes in muscle extensibility.

Sensory information from the ankle joint has been associated with the perception of verticality (Saeys et al., 2012), which is related to balance (Bonan, Guettard, Leman, Colle, & Yelnik, 2006). Successful recovery of sensory function after stroke is important for allowing the appropriate integration of sensory inputs for maintaining dynamic balance and adapting to changing environmental demands during gait (Weerdesteijn, Niet, Van Duijnhoven, & Geurts, 2008). This study showed that the expected improvements were not observed in ankle proprioception. Nevertheless, significant improvements were observed in ankle pROM, ankle strength, FML score, TUG score, BBS score, balance confidence, gait speed, and step length after 4 weeks of training. Additionally, most of the participants in the AMT-EST group said that the perception of ankle movement (from large to small ROM) and sensation of the foot area, such as the big toe, progressively improved. Another study also reported that 2 weeks of proprioception training for the big toe and ankle effectively improved light touch, postural control, and gait, but not proprioception (Lynch, Hillier, Stiller, Campanella, & Fisher, 2007). The first possible reason is that there is clearly no single precise measure of proprioception because of the complexity of the neurophysiological process (Hillier, Immink, & Thewlis, 2015). The joint position error test for ankle proprioception used during this study involves memorizing a specific target position angle and passively replicating it several times. It is inevitable that the results would be affected by the stroke patient’s mild cognition impairment related to memory, ankle muscle fatigue, and small movement error when using the equipment that we did not notice. The second possible reason is that the intensity and duration of the intervention were not sufficient.

Sensory impairments have an important role in the motor recovery and physical function of stroke patients. Depending on the lesion location, strokes can damage both the motor and sensory neural systems, block the closed loop between the brain and body, and lead to neurological impairment that is associated with significant physical dysfunction (Ang & Guan, 2013; Wieloch & Nikolich, 2006). Previously, ankle sensation has been identified as the third greatest contributor (after strength and spasticity) to gait speed after mild to moderate stroke (Hsu, Tang, & Jan, 2003). The AMT-EST group in this study particularly experienced walking speed that was significantly increased from 34.5±19.3 cm to 41.0±22.1 cm compared with that of the control group (from 33.0±22.1 cm to 37.0±26.2 cm). To successfully adapt to external cues or altered walking conditions, the central nervous system must become aware of changes in plantar pressures, limb positions, and loading. If sensory information is not integrated appropriately, then reductions in gait speed and gait asymmetry can occur (Dyer et al., 2009). It can be inferred that the significant change in step length observed in the AMT-EST group is related to the increased gait symmetry. Nevertheless, during this study, the improvement in the ankle sensorimotor function of the experimental group did not show a carryover effect, even with enhanced balance and gait, compared to the control group. In fact, the performance of complex functions, such as walking, involves various factors, including muscle strength, spasticity, cognition, motor function, and balance, as well as sensory information. A recent meta-analysis showed that leg somatosensory retraining after stroke significantly improved the somatosensory function and balance, but not gait (Chia, Kuys, & Low Choy, 2019). Synthetically, various factors, such as ankle proprioception, can be involved when considering such a complex performance.

To our knowledge, this is the first randomized, controlled trial that applied intensive passive biaxial ankle movements with EST to improve ankle sensorimotor function. This study is novel because it demonstrated the effects of AMT-EST that can improve the ankle function of chronic stroke patients who have difficulty with voluntary ankle control and its carryover effects of functional performance as a mechanism for sensorimotor integration.

This study had several limitations. First, the sample size was small. Second, because of the accidental drop-out in the control group, the number of subjects included in the analysis could be biased toward the experimental group. Third, the long-term effects of the training could not be confirmed. Fourth, we could not exclude the learning effect for each evaluation system. Finally, ankle motor control and ankle muscle activity could not be directly determined. Future studies should be performed to determine the optimal intensity and duration of this ankle intervention for participants with ankle sensorimotor impairment. Additionally, the evidence of brain plasticity for sensory recovery should be investigated.

## 5 Conclusion

This study provided evidence that AMT-EST significantly enhanced ankle pROM, strength, and functional abilities related to motor, balance, and gait functions. Therefore, AMT-EST can be part of an ankle rehabilitation program for hemiparetic stroke patients. Furthermore, ankle proprioception could be predicted by lower extremity impairment and time since stroke. Therefore, it is recommended that the lower extremity rehabilitation program should consider ankle proprioception as one of the important factors in functional performance.

## Author contributions

JE Cho: Writing - original draft preparation, study design, project administration, implementation of evaluations and interventions, formal data analysis. JH Shin: project administration, writing –review and editing. H Kim: conceptualization, methodology, study design, funding acquisition, writing –review and editing.

## Funding

This study was supported by the Translational Research Project for Rehabilitation Robots, Korea National Rehabilitation Center, Ministry of Health & Welfare, South Korea (grants #NRCTR-IN18003, 2018; #NRCTR-IN22003, 2022).

## Ethics approval

Ethics approval for this study was obtained from the Institutional Review Board at the National Rehabilitation Center on October 11, 2017. The study was registered on https://cris.nih.go.kr (unique identifier: KCT0004688).

## Conflict of interest

The authors declare that there are no conflicts of interest with respect to the research, authorship, and or publication of this article.

## Supplementary Material

Supplementary Material

## Supplementary materials

The appendices are available from https://dx.doi.org/10.3233/NRE-220018.
