# Supplementary material for: Does electrical stimulation synchronized with ankle movements better improve ankle proprioception and gait kinematics in chronic stroke? A randomized controlled study
Source: NeuroRehabilitation. 2022 Sep 30;51(2):259–69. doi: 10.3233/NRE-220018 (PMC9535592; doi:10.3233/NRE-220018)
Supplement: Supplementary Material [file nre-51-nre220018-s001.docx]

**Appendix A**

**Ankle Movement Training System**

We developed the ankle movement training (AMT) device for the quantitative measurement of ankle function and provision of rehabilitation to improve the paretic ankle function of hemiparetic stroke patients. Appendix A includes a description of the features and detailed composition of the AMT system and the ankle training protocol used during this study.

**Ankle Movement Training Device**


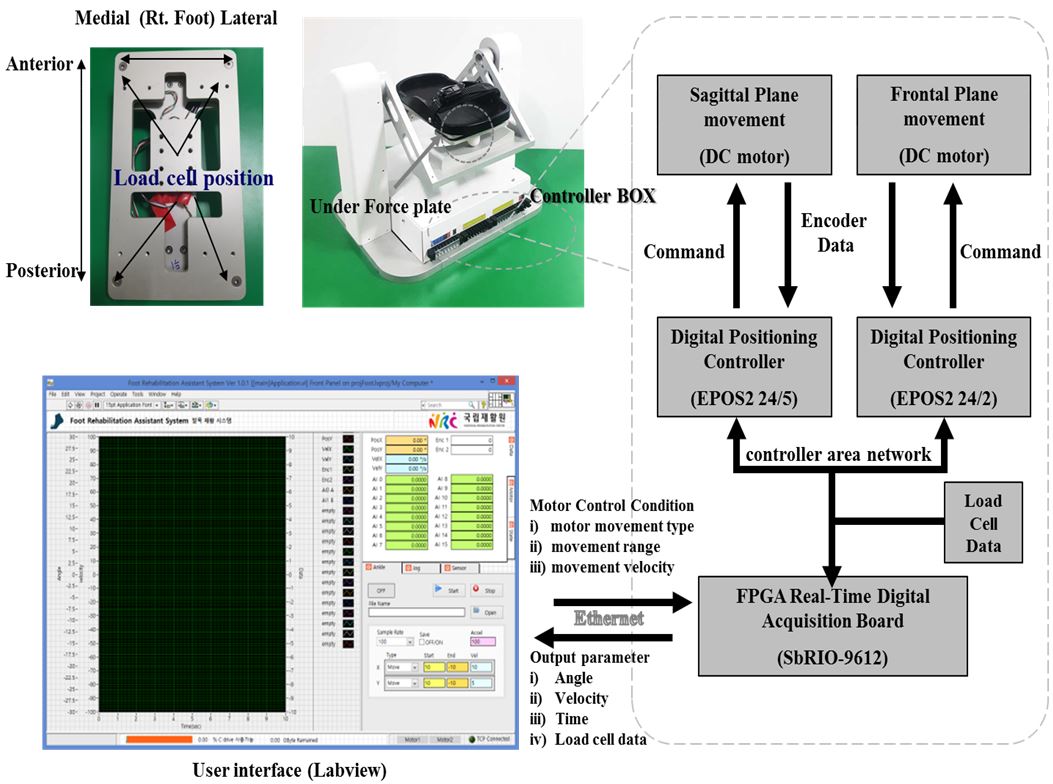


**Figure Ⅰ**. Ankle movement training hardware and software systems. Rt, right.

The AMT device consists of the force plate, foot cradle, and supporting frames (Fig. I). The ankle dorsiflexion (DF) and plantarflexion (PF) were implemented using a seesaw-type of foot cradle that pivoted along the transverse ankle axis, with the height of the malleoli determined from the surface of the footplate in the sagittal plane between two supporting side frames. For ankle supination (SN) and pronation (PN), the rear part of the foot force plate was attached to the rear supporting beam of the foot cradle and rotated along a 42-degree tilted subtalar axis relative to the foot cradle. The custom foot force plate consisted of two sandwich-paneled aluminum plates with four bar-type load cells (RS-34; Shezhen Hongrui Sensors Instrument Co., Ltd., Guangdong, China) collinearly located between four diagonal corners that were respectively fastened to the plate inside each side.

The AMT device has an interface with a control program that includes a passive/active ankle range of motion (ROM) evaluation mode and a personalized passive training mode based on the joint range setting. During training and evaluations, the foot force plate of the AMT device measured the four-quadrant (anterior, posterior, medial, and lateral) ground reaction forces sampled at 1 kHz. The first motor (Model EC-i40; Maxon Motor Inc., Sachseln, Switzerland) for the DF/PF was located around the lower supporting column, and the motor torque was transmitted via a precise timing belt and high-torque timing pulleys that rotated the foot cradle along the transverse ankle axis. The second motor for the ankle SN/PN was attached to the rear center of the foot cradle, and it transmitted the rotational torque for ankle SN and PN via a beveled gear to a 90-degree flexed axis along a 42-degree tilted subtalar axis in the sagittal plane. The movement speed along the ankle (talocrural) and subtalar (talocalcaneal) axes was 2.14 degrees/second using 14:1 high-powered planetary gearheads (GP32HP; Maxon Motor Inc.).

**Appendix B**

Assessed for eligibility (n=40)

## Enrollment

Excluded (n=5)

♦  Did not meet inclusion criteria (n=3)

♦  Other reasons (n=2)

Randomized (n=35)

## Allocation

Allocated to intervention

Experimental group (n=17)

♦ Received allocated intervention (n=17)

Allocated to intervention

Experimental group (n=18)

♦ Received allocated intervention (n=18)

## Follow-up

Lost to follow-up (n=0)

♦ Discontinued intervention (n= 0)

Lost to follow-up (n=5)

♦ Discontinued intervention: discharge (n=5)

## Analysis

Analyzed experimental group (n=12)

♦ Excluded from analysis (n=0)

Analyzed experimental group (n=18)

♦ Excluded from analysis (n=0)

**Figure Ⅱ.** Consolidated standards of reporting trials (CONSORT) diagram.

**Appendix C**

**Outcome Measures: Motor, Balance, and Gait Functions**

This study assessed the functional ability measured by motor function (the motor domain of the Fugl–Meyer Assessment of the lower extremities [FM-L]), balance (Berg Balance Scale [BBS], Timed Up and Go test [TUG], and Fall Efficacy Scale [FES]), and walking speed (10-meter walking test [10MWT]).

To measure the motor function, we used the motor domain of the FM-L.^2^ The motor domain includes measurements of movement, coordination, and reflex action of the hip, knee, and ankle. The domain is rated using a 3-point ordinal scale (0 = cannot be performed; 1 = partially performed; 2 = fully performed). The maximum possible score of the motor domain of the FM-L is 34, corresponding to full sensorimotor recovery.

To measure balance, the BBS was used as a clinical test of an individual’s static and dynamic balance.^3^ The test comprised a set of 14 simple balance-related tasks ranging from standing from a sitting position to standing on one foot. The TUG was used to assess an individual’s mobility and both static and dynamic balance.^4^ The test consists of the participant rising from the chair, walking 3 m, turning at a designated spot, returning to the seat, and sitting. The time required to perform the test is recorded using a stopwatch. A shorter time indicates good mobility and balance. The FES was applied to ascertain an individual’s level of confidence when performing activities of daily living.^5^ It is a self-report questionnaire that contains 10 items, with each scored using a scale of 0 to 10, and the total summed scores range from 0 to 100. A high score indicates high confidence when performing activities of daily living without falling.

To measure the walking speed, the 10MWT was performed.^6^ The time in seconds required to walk the middle 10-m section of a 14-m walkway was used for evaluation. The timing starts when the participant’s first foot crosses the 2-m mark, and the timing stops when the first foot crosses the 12-m mark during the course of the participant continuing to progress to the 14-m mark. An average value of the three trials was used for the analysis.

**Appendix D**

This study examined the clinical effects of the passive biaxial ankle movements that were synchronized with electrical stimulus to the corresponding ankle muscles (ankle movement training synchronized with electrical stimulation therapy [AMT-EST]). The difference between pretraining and post-training effects was properly shown by comparing the number of participants who had no proprioception during the trial when moving to 100% more of the target angle (i.e., a dorsiflexion 10-degree trial was moved to a 20-degree dorsiflexion trial, and so on) (Figure III). In the experimental group (biaxial AMT-EST group), the number of participants who had no proprioception dramatically decreased after AMT-EST. In the control group, there were fewer changes in the number of participants with no proprioception during corresponding trials because of the group participants with better proprioception. Therefore, the proposed AMT-EST could be effective for chronic stroke patients with more severely impaired proprioception.

A


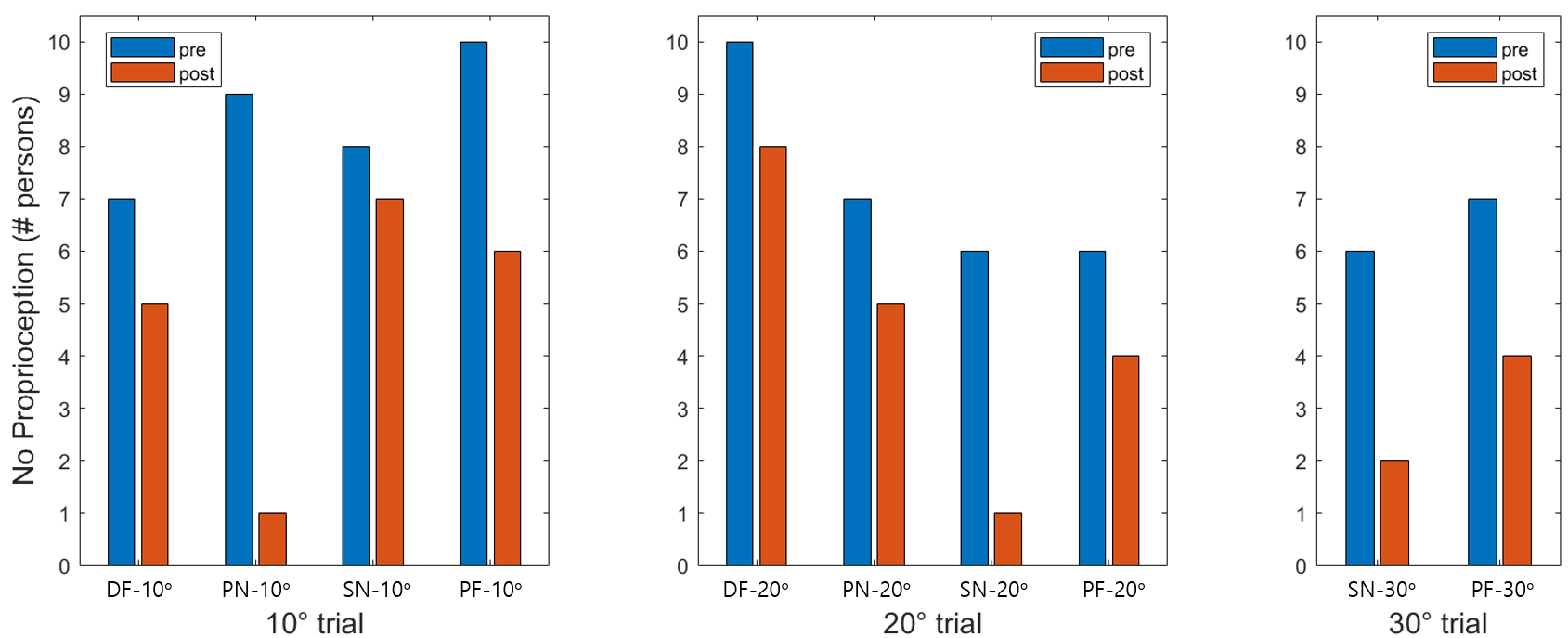


B


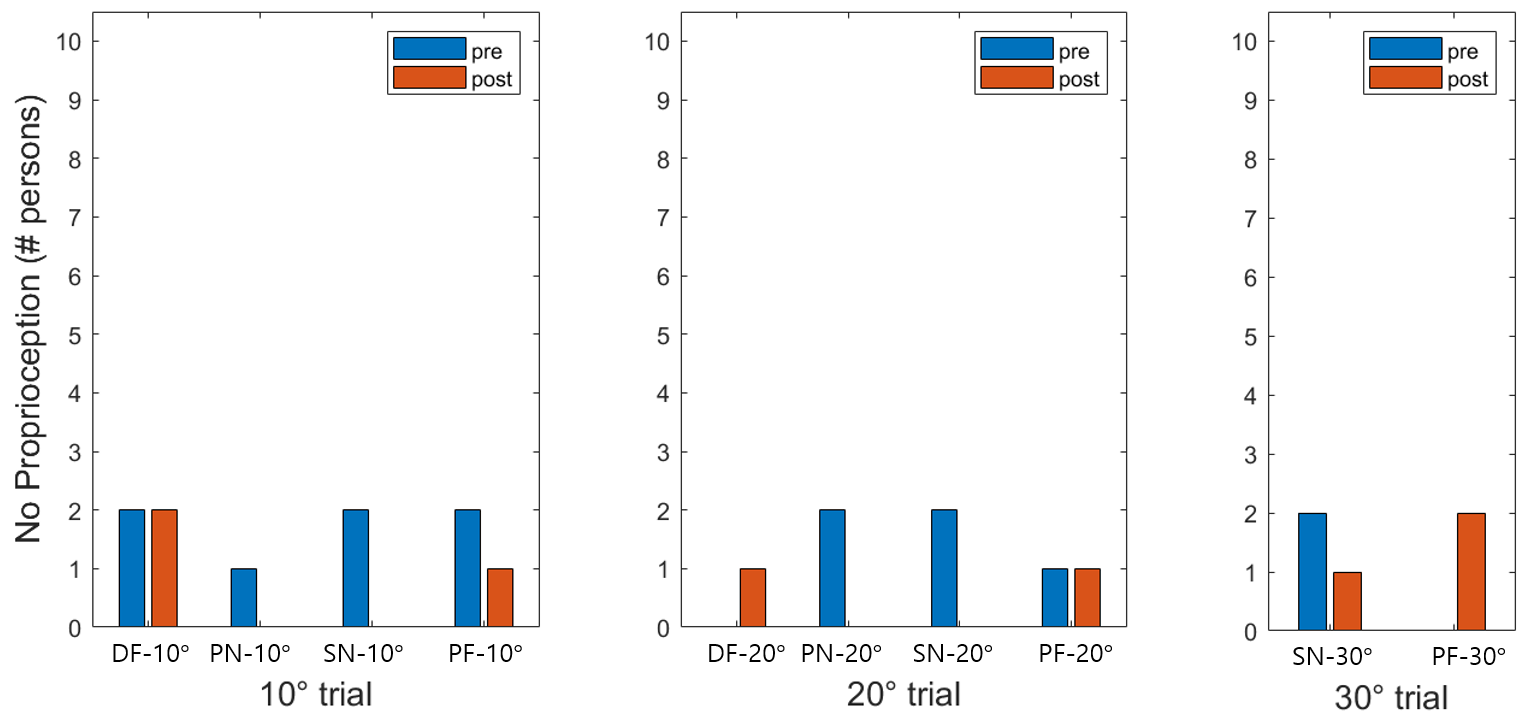


**Figure III.** Pretraining and post-training comparisons of the number of participants with no proprioception during trials. DF, dorsiflexion; PF, plantar flexion; SN, supination; PN, pronation. (A) Experimental group. (B) Control group.

**Appendix E**

This study showed that the ankle movement training synchronized with electrical stimulation therapy (AMT-EST) was effective for chronic stroke patients with more impaired ankle proprioception. The ankle proprioception was appropriately defined to show such effectiveness. To clarify the measurements, Figure IV shows boxplots and scatter plots of each ankle proprioception ratio and the corresponding values. The range of the y-axis was extended to 300% for 10-degree trials, 150% for 20-degree trials, and 100% for 30-degree trials so that each y-axis scale in the figures was matched to angles in corresponding trials.

$$Proprioception ratio=\left| \frac{Target angle-Actual angle}{Target angle} \right|$$

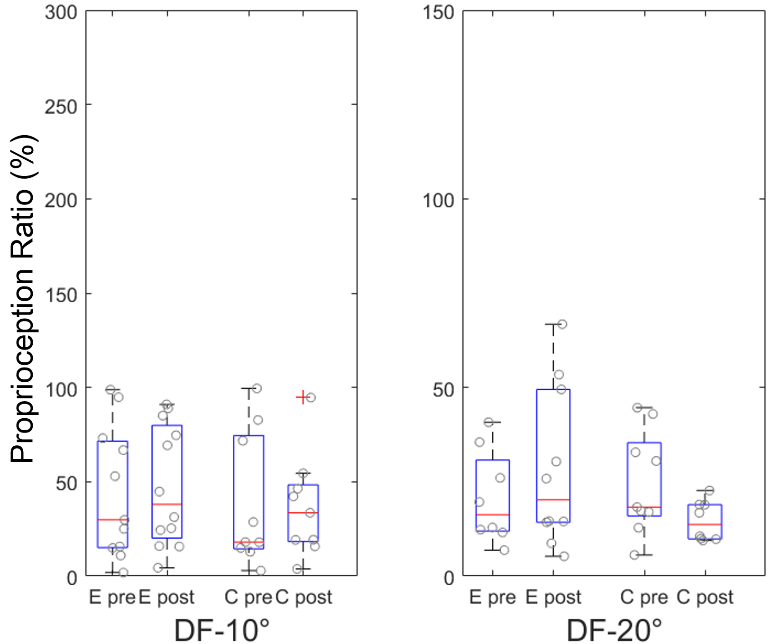


B

A

**
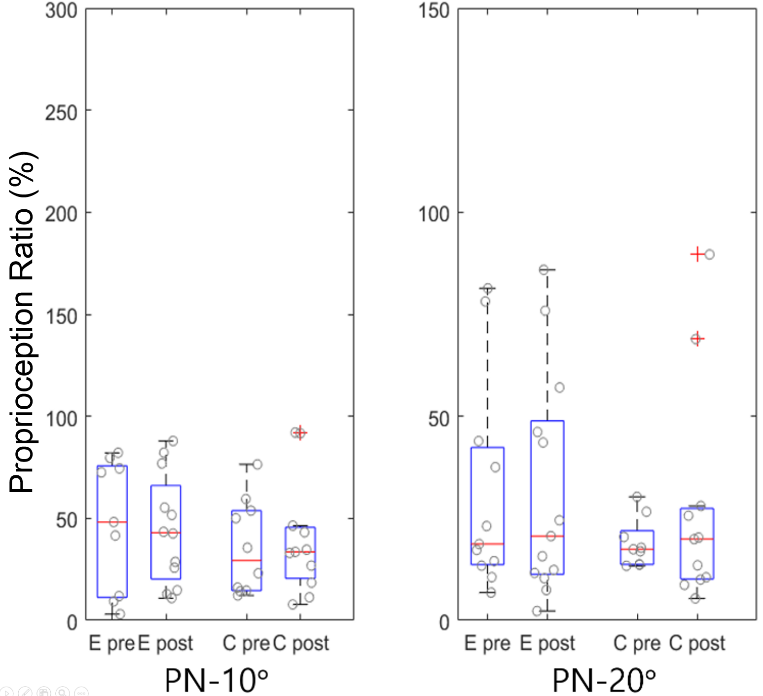
**

**
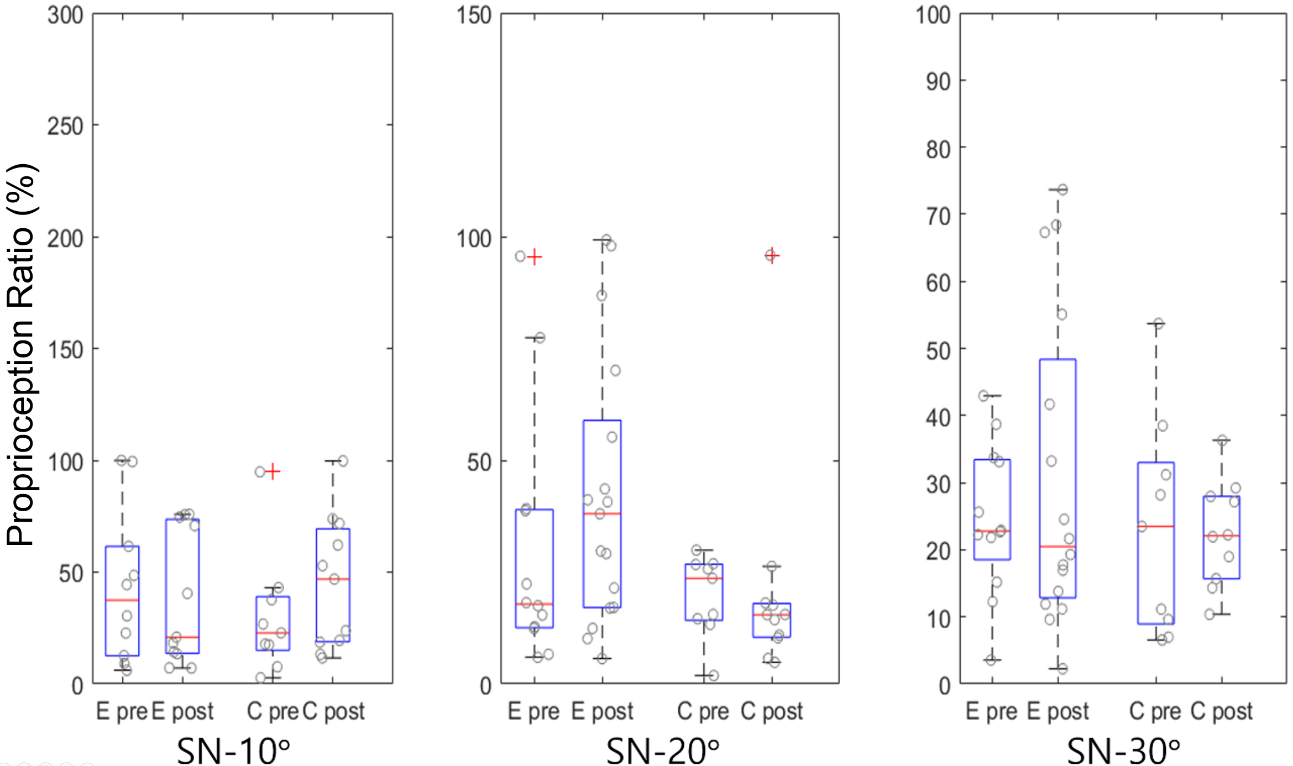
**

C

**
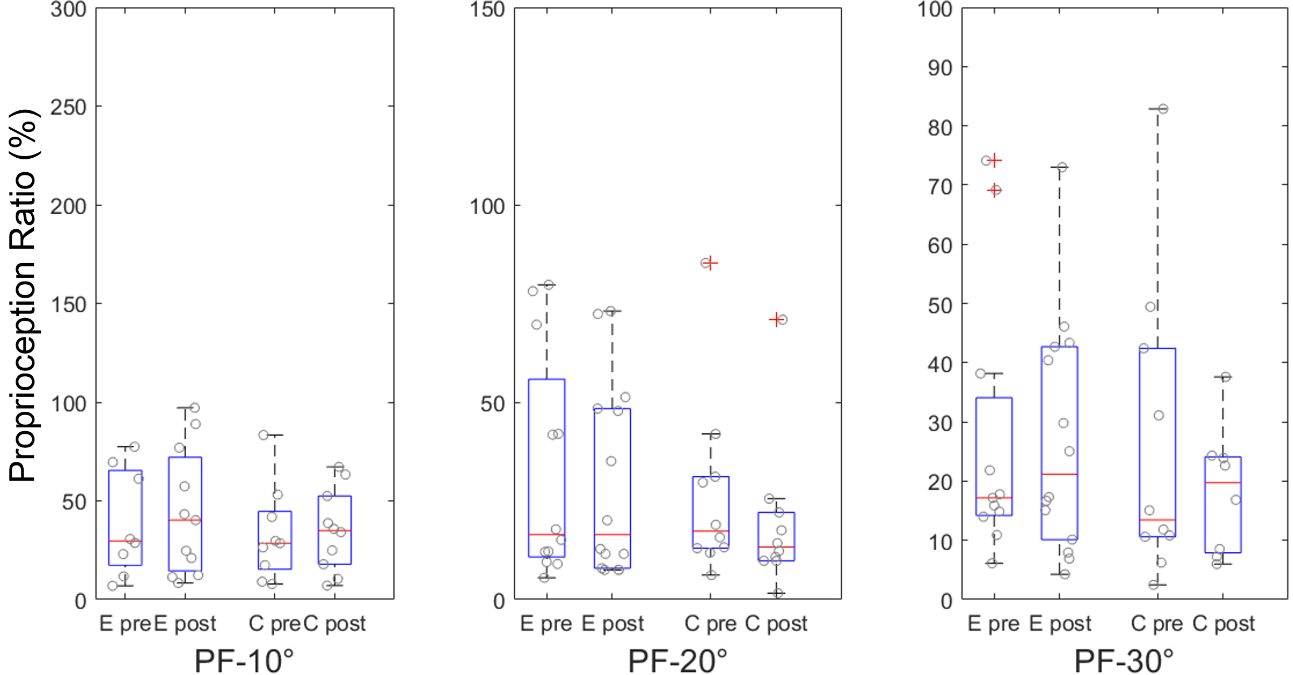
**

D

**Figure IV.** Boxplots and scatter plots of the pretraining/post-training proprioception ratios for the experimental group (denoted as E) and control group (denoted as C). (A) Dorsiflexion (DF). (B) Pronation (PN). (C) Supination (SN). (D) Plantarflexion (PF).

**Appendix F**

**Graphical abstract**
